# COVID-19 Vaccines and Assisted Reproductive Techniques: A Systematic Review

**DOI:** 10.3390/jpm13081232

**Published:** 2023-08-04

**Authors:** Elena Satorres-Pérez, Alicia Martínez-Varea, José Morales-Roselló

**Affiliations:** Department of Obstetrics and Gynaecology, La Fe University and Polytechnic Hospital, Avenida Fernando Abril Martorell 106, 46026 Valencia, Spain; elenasatorres@gmail.com (E.S.-P.); jose.morales@uv.es (J.M.-R.)

**Keywords:** COVID-19, SARS-CoV-2, vaccine, assisted reproductive techniques (ART), in vitro fertilization (IVF), reproduction, fertility, pregnancy, embryo

## Abstract

**Objective:** To review the current knowledge concerning COVID-19 vaccination and assisted reproductive techniques (ART). **Methods:** A systematic review in Pubmed-Medline, the Cochrane Database, the Web of Science, and the National Guideline was performed. Studies were selected if they were primary studies, included vaccinated (case) and unvaccinated (control) patients, and described fertility treatment response. **Results:** A total of 24 studies were selected. Outcomes related to the association between COVID-19 vaccination and ART were collected. The vast majority of studies found no statistical differences concerning oocyte stimulation response, embryo quality, implantation rates, or pregnancy outcome (clinical or biochemical pregnancy rates and losses) when comparing cases and controls. Similarly, no differences were found when comparing different types of vaccines or distinct ART (artificial insemination, in vitro fertilization, and embryo transfer of frozen embryos). **Conclusions:** Patients receiving ART and health care professionals should be encouraged to complete and recommend COVID-19 vaccination, as the available evidence regarding assisted reproductive outcomes is reassuring.

## 1. Introduction

SARS-CoV-2 is a single, positive-strand RNA virus [1]. Until the first identification of human coronaviruses in the 1960s, coronavirus infections were known to be inoffensive to humans [2]. Since then, the total number of human coronaviruses identified has increased throughout the years. While most infections cause minor respiratory symptoms, some may cause major problems, especially in high-risk patients such as the elderly, infants, immunodeficient patients, and individuals with chronic respiratory diseases [3,4].

SARS-CoV-2 was first identified in Wuhan (China) in late 2019. By the end of January 2020, 7734 cases had been confirmed in China, and 90 other cases had also been reported in 19 different countries. The mortality rate was estimated to be 2.2% [5]. According to the World Health Organization, by 19 July 2023, there will have been 768,237,788 confirmed cases of COVID-19, including 6,951,677 deaths reported [6].

Since the declaration of the COVID-19 pandemic in March 2020, researchers have aimed to answer healthcare professionals’ and patients’ questions concerning the infection: its origin, transmission, mechanism, and management.

To control the sanitary crisis, diverse vaccines were developed worldwide. The FDA has approved four formulations and has helped, through massive vaccination, to constrain the pandemic [7]. According to the World Health Organization, as of 23 July 2023, a total of 13,474,348,801 vaccine doses had been administered [6]. It is interesting to discern the different kinds of developed vaccines and their mechanisms of action:

1. Inactivated vaccines have been widely used and are well-established for infections such as influenza [8] and poliovirus [9]. These vaccines are produced by disarming pathogens through heat, radiation, or chemicals such as formalin or formaldehyde in order to maintain their immunogenicity but block their capability to replicate and infect [10].
2. Quite the reverse, COVID mRNA vaccines are the first to be approved with this formulation. They use the host’s own cellular function to synthesize a viral protein product and establish protective immunity [11,12].
3. Recombinant subunit vaccines contain fragments (proteins, polysaccharides, etc.) of the pathogen. These parts are enough to trigger the host’s immunity, although they could be less immunogenic than other types of vaccination. Examples of subunit vaccines are hepatitis B, clostridium tetani, or papillomavirus [10].
4. Non-replicating viral vector vaccines use an innovative approach to create host immunity. They use the capacity of other viruses with heterologous antigens to infect cells and induce an antigen-specific humoral and cellular immune response [13].

The global sanitary crisis aroused many questions and uncertainties about the long-term consequences and adverse effects of suffering from the infection. Additionally, the rapid development of the above-mentioned vaccines awakened distrust in a large part of the population. Fertility treatment patients were not to be an exception; it is widely known that fertility and reproductive treatments constitute a stressful event for most patients who have difficulties achieving pregnancy [14,15]. During the initial phases of the pandemic, many fertility treatments were postponed, with a subsequent psychological impact on women and their partners and an increase in their stress and anxiety [16,17,18].

Despite the directions provided by international organizations, physician and patient hesitancy towards vaccination during pregnancy and preconception has remained an issue. Some studies report that <50% were willing to get vaccinated during these periods [19,20]. Published surveys and inquiries suggest that around 30% of infertile couples undergoing assisted reproductive treatment delayed the primary vaccination [21]. 

Nevertheless, according to the Centers for Disease Control and Prevention, in 2020, there were 326,468 ART cycles performed at 449 reporting clinics in the United States, resulting in 75,023 live births (deliveries of one or more living infants) and 79,942 live-born infants [22]. Comparing these ciphers to those reported in 2019, it is evident that COVID did not impede infertile patients from initiating ART. 330,773 new cycles were reported in 2019, of which 95,030 resulted in pregnancies, 77,998 live-birth deliveries (delivery of one or more living infants), and 83,946 infants [23]. It is evident that infertility treatments did not decrease despite the COVID-19 sanitary crisis; however, it probably stopped thousands of individuals from accepting and receiving vaccination. 

Some of the main fears arose from uncertainty: Could COVID-19, prior to or during assisted reproductive techniques (ART), negatively influence the results of the treatment? Does COVID-19 vaccination during ART have deleterious effects on the treatment? What if previous immunity to SARS-CoV-2—through infection or vaccination—influences ART success?

Published investigations, as well as previous systematic reviews and meta-analyses, have aimed to answer these questions and clarify the association between COVID-19 and fertility. Many investigators defend that sperm production can be altered, as male gonads can be vulnerable to infection [24,25,26]. This can be explained by the presence of angiotensin-converting enzyme 2 (ACE2) receptors, which are abundant in testes, seminiferous duct cells, spermatogonia, Leydig, and Sertoly cells [27,28]. It is known that SARS-CoV-2 enters the host cell by ligating precisely to these ACE2 receptors. Furthermore, fever is one of the most frequent symptoms among those suffering from COVID-19. This state can cause dysregulation of sperm production and development, as a stable scrotal temperature is essential for this process [29].

In addition, numerous published studies to date have questioned if COVID-19 could also affect female fertility and disrupt its functions. ACE2 receptors are also present in endometrium cells and are involved in follicular and ovulation regulation and development, angiogenesis, and luteal degeneration [27]. Furthermore, the cytokine storm and consequent appearance of reactive oxygen species secondary to inflammation and the immune response activated by COVID-19 infection could cause ovarian damage and disrupt oocyte development and normal embryo implantation [30]. Nevertheless, most studies conclude that no evidence suggests COVID-19 infection could significantly alter female fertility [31].

Nevertheless, there is scarce evidence concerning COVID-19 vaccination and fertility, and valuable data is even harder to find referring to patients undergoing ART. This review aims to summarize the existing evidence concerning this group of patients to resolve some of the unanswered questions and guide healthcare professionals and patients in their decision-making. 

## 2. Methods

### 2.1. Information Sources

This systematic review was developed following the Preferred Reporting Items for Systematic Reviews and Meta-Analyses (PRISMA) guide. Publications from the following sources were included: Pubmed-Medline, Cochrane Database, Web of Science, and National Guideline. 

### 2.2. Eligibility Criteria

All observational and experimental studies concerning the COVID-19 vaccine and ART were considered adequate for this review. Subsequently, exclusive criteria were applied: (1) secondary studies (reviews, metaanalyses), (2) studies not measuring ART outcome, (3) studies not evaluating COVID-19 vaccination, and (4) studies without full text available.

As the present article is a systematic review, only primary investigations have been analyzed. Other secondary sources, such as systematic reviews and meta-analyses, are included and debated in the discussion section.

### 2.3. Search Strategy

In order to define our search, a PICO question was defined: P (patient = patient undergoing ART), I (intervention = COVID-19 vaccines), C (comparison = not vaccinated), and O (outcome = fertility treatment response). Then, MeSH terms were defined: COVID-19, vaccine, ART, in vitro fertilization (IVF), embryo, SARS-CoV-2, and coronavirus.

### 2.4. Data Collection Process

**Figure 1:**
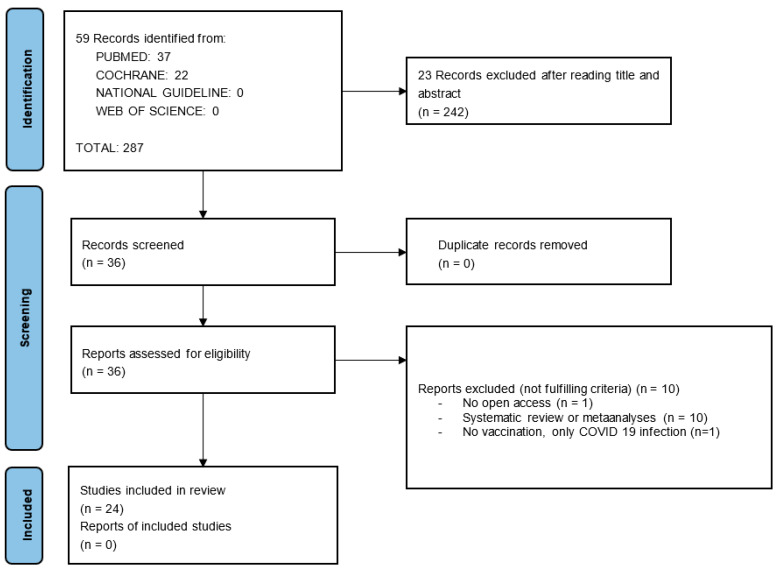
Flow diagram representing the article selection process.

The literature search was executed carefully according to PRISMA guidelines [32]. Formerly, titles and abstracts of all articles were screened to exclude articles that were not focused on the theme, and subsequently, an intense lecture on each article was performed. Thus, potential exclusion criteria were detected. Finally, a discussion was conducted between collaborators, and a final selection was made by consensus. The selection process is shown in the following flow diagram, Figure 1.

### 2.5. Bias Assessment

The described eligibility criteria were strictly defined and applied to reduce the risk of bias in our systematic review. The research was based on multiple information sources, including English, French, and Spanish articles. This scrutiny was performed in an objective and reproducible manner. Furthermore, and as previously detailed, two authors analyzed and screened the selected articles to contrast and refine the final selection. 

## 3. Results

### 3.1. Search Results

A total of 59 articles were initially collected from four different databases. Twenty-three records were excluded after reading the title and abstract, as the main subject was unrelated to the investigated matter. Thus, 36 reports were assessed for eligibility and submitted to an intense evaluation. Finally, 24 documents were selected to constitute this review after excluding 12 articles for not meeting the inclusion criteria. Figure 1 details the justifications for article exclusion. 

### 3.2. Characteristics of Included Studies

Altogether, 3469 ART cycles were gathered and analyzed. Control groups consisting of unvaccinated patients summed up to 15,124 individuals, while cases were divided between vaccinated patients (n = 6656), those with a passed COVID-19 infection (n = 71), and those accomplishing both situations (n = 34). The mean total sample size was 143,204 patients per article; however, it should be highlighted that the sample size was rather discordant (from 32 up to 10,541). Most of the included studies had retrospective cohort designs (66.6%), while the rest displayed prospective cohort designs. Furthermore, most were single-centered studies (75%) based on six different countries: China (11), Israel (6), Spain (3), the United States of America (2), Jordan (1), and Austria (1). 

All the selected articles except three, detailed the type of vaccine administered to the included patients. The most prevalent vaccine among the included sample was the inactivated virus vaccine (50%), followed by messenger RNA (mRNA) vaccines (41.67%), adenovirus vector vaccines (20.8%), and finally recombinant subunit vaccines (12.5%). Only five studies compared results between different vaccines, as discussed further [33,34,35,36,37]. 

Regarding ART techniques, most studies analyzed IVF cycles (83.3%). A minority included data concerning embryo transfer (ET) exclusively (16.6%) and artificial insemination (AI) procedures (4.2%).

Detailed study characteristics are described in Table 1. Table 2 shows the variables that were assessed in the included studies.

### 3.3. Ovarian Stimulation and Oocyte Retrieval

Most studies analyzed the number of oocytes retrieved (n = 17) and their quality (n = 14). The vast majority of studies agree that no evidence was found to suggest that COVID-19 vaccination negatively affects cycle stimulation characteristics, as no differences between case and control groups were detected in any of the surrogate parameters for ovarian follicle quality [34,36,38,40,41,46,47,48,49,50,51,52,53,54,56].

Nevertheless, one Spanish study published in 2023 revealed a higher number of oocytes retrieved in vaccinated patients, though their quality was significantly lower [42]. Similarly, a Chinese study including 536 patients undergoing IVF/intracytoplasmatic sperm injection (ICSI) found lower-quality oocytes in vaccinated women [33]. 

### 3.4. Fertilization, Embryo Development, and Transfer

A total of 12 studies evaluated the number of fertilized oocytes. The most common finding was that COVID-19 vaccination did not affect this parameter [34,36,40,46,49,50,51,52,53,56], although two other of the included studies differed [33,47]. 

A Chinese study including women who received inactivated or recombinant COVID-19 vaccination obtained lower fertilization rates in women who had received the recombinant vaccine, especially in those who received ART treatment less than six weeks after vaccination [47]. This, however, did not substantially affect the primary study endpoints: average fertilization rate and clinical pregnancy rate. On the contrary, an American study displays that in their studied sample, vaccinated patients had higher mean and standard deviation (SD) fertilization rates than unvaccinated patients (77.45% (41.45%) vs. 68.66% (20.51%); *p* = 0.03) [33].

Regarding embryo quality and euploidy, only two studies have found significant differences between groups. Dong et al. [34] affirm no significant differences in laboratory results (high-quality embryo rate and blastocyst formation rate) among groups (*p* > 0.05). Nonetheless, the embryo transfer stage (cleavage or blastocyte) and type (fresh or frozen) did have significant differences among the four groups: a higher rate of cleavage vs. blastocyst was found among groups with both partners receiving two doses of COVID-19 vaccine or neither of them vaccinated compared to groups receiving only one of the members of the couple vaccinated. Contrarily, Jacobs et al. describe that the number of embryos at the cleavage stage was significantly lower in the vaccinated group; however, with no significant differences in pregnancy rates [47]. The rest of the included studies revealed that vaccination status did not affect embryo quality [33,36,38,39,42,44,45,46,47,50,52,53,56] or euploidy [38,46]. 

Nine studies have evaluated the number of embryos transferred [33,34,39,40,44,45,46,50,52,53]. Two studies detail those available for posterior cryopreservation [40,47], and another study points out the state of each embryo when transferred [50]. All studies conclude that there is no evidence to suggest that COVID-19 vaccination negatively affects these embryological variables.

### 3.5. Implantation, Pregnancy, and Loss

It is convenient to clarify the definition of “implantation rate,” meaning the total number of early gestational sacs/the total number of transferred embryos × 100%. Besides, the clinical pregnancy rate is the number of pregnancy cycles/the total number of transfer cycles × 100%. 

All ten studies that assessed implantation rates agree that no statistically significant differences were found when comparing couples with completed vaccinations vs. those unvaccinated [33,34,35,40,42,43,45,46,52,53,55]. Equally, clinical pregnancy rates and data concerning ongoing pregnancy, biochemical loss, and clinical loss support that COVID-19 vaccination is safe and effective and has no impact on fertility [33,34,38,39,40,41,42,43,44,45,46,47,49,50,51,52,53,55,56].

### 3.6. Comparison between Vaccines

Five articles compare the results of different types of vaccines, Table 3. Among them, only one found statistical differences when comparing inactivated virus vaccines and recombinant subunit vaccines regarding oocyte maturation and fertilization rates [33]. 

## 4. Discussion

The global COVID-19 pandemic is currently under control, given that the WHO decreed the end of the international emergency due to COVID on 5 May 2023 [57]. Massive vaccination has indeed helped to dominate the crisis, as COVID-19 vaccination has proved not only to protect against severe symptoms of the disease but also to be an essential tool in decreasing the spread of the virus and the rate of infection [58,59,60].

Many studies have already demonstrated high security in vaccination during pregnancy [61,62] and vaccinated women with SARS-CoV-2 infection are associated with decreased hospital admission due to COVID-19 as well as reduced progression to severe COVID-19 [63]. 

This systematic review concerning COVID-19 vaccination and ART shows excellent vaccine safety evidence for couples requiring ART. The vast majority of studies are consistent about unaltered results in oocyte maturation and retrieval, fertilization, embryo development and transfer, implantation, pregnancy, and loss. 

The gathered information suggests that all evaluated vaccines (inactivated, adenovirus vector, recombinant, and mRNA) constitute a safe option, with no significant differences in oocyte stimulation response, embryo quality, or pregnancy outcomes. This is applicable among infertile couples in which both, only one, or neither of the partners were vaccinated against COVID-19.

Three systematic reviews and meta-analyses concerning COVID-19 and ART results have been previously published. Chen et al. [33] thoroughly reviewed the literature and compared 15 studies on the safety of COVID-19 vaccines in 536 women undergoing fresh embryo transfer after IVF/ICSI treatment. Coinciding with the findings of the present systematic review, they concluded that implantation rates were not significantly lower in vaccinated patients.

Likewise, a systematic review and meta-analysis published in April 2023 analyzed 18,877 individual cases undergoing IVF [64], vaccinated with either an mRNA or inactivated virus vaccine. Their results conclude that vaccination against COVID-19 did not adversely affect the different stages of the process (number of oocytes and MII/mature oocytes obtained; implantation, blastocysts, and fertilization rates) or the final outcome (biochemical pregnancy rates) of IVF. 

A third article evaluates ART cycle results before and after the COVID pandemic by analyzing seven previous studies of 33,883 ART cycles [65]. The findings of this review describe that no statistical differences were found before and after the COVID-19 pandemic for all of the studied outcomes (both clinical and laboratory). Nevertheless, it is essential to highlight that this review does not assess vaccination status but evaluates whether the COVID-19 infection itself and the subsequent sociosanitary changes secondary to the pandemic state (such as quarantine measures and changes in the frequency of medical visits) could affect the laboratory and clinical outcomes of women undergoing ART.

The present systematic review detected several limitations in the gathered evidence. Firstly, only observational studies have been found, as no placebo-controlled studies in vaccinated patients have been developed. Future investigations should design, in this manner, higher-quality studies in the furtherance of strengthening the present evidence. Furthermore, paternal factors are barely evaluated, despite being an essential element in fertility and ART. This factor should be solidly considered and treated as confounding if only female vaccination or immunity were assessed. In addition, only five studies compared different types of vaccines. However, none included all varieties, and limited evidence is published concerning ART techniques rather than IVF (IA, timed intercourse, ovulation induction techniques). Similarly, long-term pregnancy outcomes are extremely scant. Many studies have proven the safety of COVID-19 vaccines before conception and during pregnancy [66]. However, ART pregnancies are not studied independently. All these aspects need to be approached and deserve attention. 

To the best of our knowledge, this is the first systematic review concerning COVID-19 vaccination and ART, including the four types of vaccines developed. An ulterior meta-analysis would strengthen the assembled evidence and give a clearer view of the subject.

In conclusion, this systematic review reveals that there is no scientific evidence of any association between COVID-19 vaccination and adverse outcomes in ART. Thus, patients and healthcare professionals should be reassured about completing vaccination schedules before starting ART. Nonetheless, more data is warranted to confirm that long-term pregnancy outcomes are not altered.

## Figures and Tables

**Table 1:** Characteristics of the included studies.

Article Details				Sample (Cycles)					Type of Vaccine				ART Technique		
Article	Country and Date of Publication	Centre	Design	Cases	Passed COVID-19	Vaccinated	Passed and Vaccinated	Control	Inactivated Virus	mRNA	Adenovirus	Recombinated	IVF	ET	AI
Aharon et al. [38]	EEUU, 2022	Single centre	Retrospective cohort study	1205	0	222	0	983		x			x		
Aizer A et al. [39]	Israel, 2022	Single centre	Retrospective cohort study	672	44	220	0	408		x			x		
Albeitawi S et al. [40]	Jordan, 2022	Multicentric study	Retrospective cohort study	151	18	66	34	33	x	x	x		x		
Avrahan S et al. [41]	Israel, 2022	Multicentric study	Retrospective cohort study	400	0	200	0	200		x			x		
Bosch A et al. [42]	Spain, 2023	Multicentric study	Retrospective cohort study	230	0	115	0	115		x			x		
Brandao P et al. [43]	Spain, 2022	Multicentric study	Retrospective cohort study	4162	0	890	0	3272		x			x		
Cao M et al. [44]	China 2022	Single centre	Retrospective cohort study	2101	0	5’2	O	1589	x				x		
Chen H et al. [33]	china 2023	Single centre	Retrospective cohort study	536	0	268	0	268	x			x	x		
Dong M et al. [34]	China, 2022	Single centre	Prospective cohort study	735	0	221	0	514	x		x	x	x	x	
Huang J et al. [45]	China 2022	Single centre	Retrospective cohort study	1210	0	265	0	265	x					x	
Huang J et al. [46]	China 2022	Single centre	Retrospective cohort study	133	0	66	0	67	x				x		
Jacobs E et al. [47]	EEUU, 2022	Single centre	Prospective cohort study	280	0	142	0	138						x	
Karavani G et al. [48]	israel, 2022	Single centre	Retrospective cohort study	138	0	83	0	55		x			x		
Odeh-Natour R et al. [49]	Israel, 2022	Single centre	Prospective cohort study	59	0	37	0	22					x		
Orvieto R et al. [50]	Israel, 2021	Single centre	Prospective cohort study	36	0	36	0	36		x			x		
Requena A et al. [36]	Spain, 2023	Multicentric study	Retrospective cohort study	1700	0	510	0	1190		x	x		x		
Chillon T et al. [51]	Austria, 2023	Single centre	Prospective cohort study	89	0	45	0	44					x		
Wang C et al. [35]	China 2022	Multicentric study	Prospective cohort study	4185	0	2129	0	2056	x		x	x			x
Xia W et al. [52]	China 2022	Single centre	Prospective cohort study	260	0	105	0	155	x				x		
Wu Y et al. [53]	China 2022	Single centre	Retrospective cohort study	240	0	0	0	1343	x				x		
Bentov Y et al. [54]	Israel, 2021	Single centre	Prospective cohort study	32	9	9	0	14		x			x		
Wang Y et al. [55]	China 2022	Single centre	Retrospective cohort study	1496	0	460	0	1036	x					x	
Yin J et al. [56]	China 2023	Single centre	Retrospective cohort study	10,541	0	835	0	1670	x		x		x		
Zhao Yan et al. [37]	China 2022	Single centre	Retrospective cohort study	3778	71	728	34	3050	x						

Assisted reproductive techniques (ART), embryo transfer (ET), and artificial insemination (AI).

**Table 2:** Variables studied in the included studies. * SD: statistical differences.

Article	Country and Date of Publication	Endometrial Thickness	Number of Oocytes Retrieved	Quality of Oocytes	Number of Fertilized Oocytes	Number and Quality of Embryos	Number of Embryos Transferred	Number of Frozen Embryos	Euploidy	Implantation Rate	Clinical Pregnancy Rate	Ongoing Pregnancy	Biochemical Loss	Clinical Loss	Other
Aharon et al. [38]	EEUU, 2022		NS	NS		NS			NS		NS	NS	NS	NS	
Aizer A et al. [39]	Israel, 2022	NS				NS	NS				NS	NS	NS	NS	
Albeitawi S et al. [40]	Jordan, 2022	NS	NS	NS	NS	Lower in vaccinated	NS	NS		NS	NS				
Avrahan S et al. [41]	Israel, 2022		NS								NS				
Bosch A et al. [42]	Spain, 2023		Higher in vaccinated	Lower in vaccinated		NS				NS	NS				
Brandao P et al. [43]	Spain, 2022									NS	NS				
Cao M et al. [44]	China, 2022	NS				NS	NS				NS	NS	NS	NS	Ectopic, brith height and weight
Chen H et al. [33]	China, 2023	NS	NS	Lower in vaccinated	Lower in vaccinated	NS	NS			NS	NS			NS	
Dong M et al. [34]	China, 2022	NS	NS	NS	NS	* SD in 4 groups	NS	NS		NS	NS				
Huang J et al. [45]	China, 2022	NS				NS	NS			NS	NS		NS		Birth
Huang J et al. [46]	China, 2022		NS	NS	NS	NS			NS	NS	NS		NS		
Jacobs E et al. [47]	EEUU, 2022		NS	NS	Higher in vaccinated	NS		NS			NS	NS		NS	
Karavani G et al. [48]	Israel, 2022		NS	NS											
Odeh-Natour R et al. [49]	Israel, 2022	NS	NS	NS	NS						NS				
Orvieto R et al. [50]	Israel, 2021		NS	NS	NS	NS	NS				NS				
Requena A et al. [36]	Spain, 2023		NS	NS	NS	NS									
Chillon T et al. [51]	Austria, 2023		NS		NS						NS	NS	NS	NS	Birth
Wang C et al. [35]	China 2022										NS				
Xia W et al. [52]	China, 2022		NS	NS	NS	NS	NS			NS	NS	NS			Sperm quality, NS
Wu Y et al. [53]	China, 2022	Lower in vaccinated	NS		NS	NS	NS			NS	NS	NS	NS	NS	Ectopic
Bentov Y et al. [54]	Israel, 2021		NS	NS											Igg in folicular fluid, NS
Wang Y et al. [55]	China, 2022	NS								NS	NS			NS	Ectopic
Yin J et al. [56]	China 2023		NS	NS	NS	NS					NS	NS			
Zhao Yan et al. [37]	China 2022										NS			NS	

**Table 3:** Comparison between different types of vaccines.

Article	Country and Date of Publication	Vaccine			
		Inactivated Virus Vaccines	ADENOVIRUS Vector	Recombinant Subunit	mRNA
Dong M et al. [34]	China, 2022	NS	NS	NS	
Chen H et al. [33]	China, 2023	Lower oocyte maturation and fertilization rate		NS	
Wang C et al. [55]	China, 2022	NS	NS	NS	
Requena A et al. [36]	Spain, 2023		NS		NS
Yin J et al. [56]	China, 2023	NS	NS		

## Data Availability

Not applicable.
